# Molecular network analysis of 308 newly diagnosed HIV infection and 210 ART failure patients from rural counties in Sichuan

**DOI:** 10.1371/journal.pone.0298324

**Published:** 2024-02-16

**Authors:** Xia Zhong, Dan Yuan, Shuang feng Fan, Yang Liu, Ling Su, Shi Jiao He, Shu Liang, Yi Yang

**Affiliations:** 1 School of Management, Chengdu University of Traditional Chinese Medicine, Chengdu, China; 2 Institute of HIV/AIDS prevention, Sichuan Center for Disease Control and Prevention, Chengdu, China; 3 Department of HIV/AIDS prevention, Chengdu Center for Disease Control and Prevention, Chengdu, China; Nanyang Technological University, SINGAPORE

## Abstract

**Background:**

Few studies on molecular epidemiology have studied people with newly diagnosed HIV infection and ART Failure Patients at the same time in rural China. With more serious HIV epidemic than in other provinces in China, Sichuan is an area suitable for this study.

**Objective:**

To analyze the characteristics of HIV-1 molecular networks and factors related to network entry among newly diagnosed HIV infection and ART Failure Patients in three county-level cities (A, B, C) in Sichuan Province, to provide scientific basis for accurate prevention and control.

**Methods:**

Nested PCR amplification method was used to amplify HIV-1 pol gene region of 530 blood samples, Sequencer 4.9 was used to edit, clean and splice the gene sequence, Bioedit correction, Fastree 2.1.8 and Figtree 1.4.2 to construct evolutionary tree and determine genotype. HyPhy2.2.4 and Cytoscape 3.6.1 software were used to construct molecular network. Logistic regression analysis was applied.

**Results:**

523(98.68%) pol sequences were obtained, and a total of 518 valid sequences with basic information came into the final analyses. A total of 6 genotypes were detected, namely CRF01_AE (320,61.78%), CRF07_BC (149,28.76%), B (30,5.79%), CRF08_BC (11, 2.12%), CRF55_01B (6, 1.16%) and C (2, 0.39%). 186 of 518(35.91%) sequences entered the network at a genetic distance of 0.8%, forming 42 propagation clusters. “High-risk transmitters”(connected with two and more) accounted for 21.62%. Logistic regression showed that≥50 years old (OR = 2.474) were more risky than 18–49 years old, CRF07_BC sub-type (OR = 0.174) were less risky than CRF01_AE sub-type, B sub-type (OR = 6.698) is higher risky than CRF01_AE sub-type, and District B (OR = 0.077) less risky than that of A city.

**Conclusion:**

The sources of HIV infection in rural Sichuan are diversified and complicated. The prevention and control of HIV infection in Sichuan Province should focus on strengthening the long-term dynamic detection of elderly population, B strain sub-type, and in City A.

## Introduction

China is facing big challenges to ending the Human Immunodeficiency Virus/Acquired Immune Deficiency Syndrome (HIV/AIDS) epidemic as a public health threat [1, 2] as in the world [3]. The HIV epidemic in China is unevenly distributed in time and space, and the epidemic in Sichuan Province is more serious than in other provinces [2]. Heterosexual transmission have been prominent in HIV epidemic in China [1], but the role of homosexual transmission at population level is not clear yet in China. Chengdu is the capital city of Sichuan Province. Besides the total increasing number of HIV/AIDS [1], another challenge for HIV prevention in Chengdu is dynamic men who have sex with men(MSM) and high HIV prevalence among them. The overall HIV prevalence among MSM in Chengdu between 2009 and 2014 was 15.5% [4]. Between 2012 and 2018, the HIV incidence density among MSM decreased annually, but the total incidence density was as high as 5.95 (95% CI: 5.37–6.56)/100 person-years [5]. Moreover, in 2013, 17.9% of MSM reported that they had sex with both men and women(MSM/W), and living in Chengdu was found out as risk factor [6].

In 2019, the Chinese Center for Disease Control and Prevention (CDC) issued the "HIV Transmission Network Monitoring and Intervention Technical Guidelines (Trial)", proposing that through investigation and analysis based on HIV-1 molecular networks, high-risk transmitters and groups can be detected in time, and precise interventions can be applied to reduce newly diagnosed HIV infection. The Sichuan Provincial People’s Government issued the "Implementation Opinions on Promoting Healthy Sichuan Actions" and stated that it is necessary to strengthen HIV source control, monitoring and testing to curb HIV epidemic and reduce its harm effectively. In 2020, 10 provincial government departments including the Sichuan Provincial Health Commission jointly formulated the "Sichuan Province Implementation Plan for Containing the Spread of AIDS (2020–2022)"(Implementation Plan). According to the Implementation Plan, newly diagnosed HIV infection, drug resistance monitoring, and CD4+ T cell detection should be combined, so that key populations and areas with newly diagnosed HIV infection could be accurately identified, and effectively targeted preventive measures could be taken. Studies have shown that if one patient continues to carry a high level of viral load, its CD4+ T cell count will continue to decrease and result in a decrease in body immunity and an increase in infectivity [7, 8].

Constructing HIV-1 molecular networks and conducting molecular network analysis are effective methods to give biological non-bias evidence for HIV transmission, which provides a scientific basis for studying the origins and spread of HIV/AIDS, and also provides new ideas for precise intervention and control [9, 10]. For example, based on the results of molecular network analysis of 40,950 sequences during 2001–2012 at national level, HIV infection among heterosexual women has been proven to be likely originating both from MSM and heterosexual men in US for the first time [11]. In 2015, a molecular network analysis of 16 male intravenous drug users(IDU) and their heterosexual partners found out that 14 pairs of genes were close to less than 0.015 indicating that there were direct infection relationships, then epidemiological investigations confirmed the existences of contagious relationships [12]. In the same year, Chinese scholars collected 324 nationwide HIV infected blood samples from 1996 to 2006 whose infection route was unknown, they performed genetic sequencing and measured the gene of the virus strain. Finally, 100 of the 225 samples were identified being infected through blood transmission, 114 by injection, and 11 through sexual transmission [13]. In 2018, a German scientific research team reported that using molecular network transmission structure of the Cologne region, they found that central Cologne may be a concentrated HIV epidemic area [14].

In summary, molecular network analysis has been used to carry out HIV traceability investigation, and epidemiological investigation has been used to confirm suspicion. Studies from rural areas where residents travel less than in the cities are easy to construct transmission network [15]. Most studies on molecular networks are mainly based on the study of the transmission characteristics of the molecular networks of HIV infections or the detection of drug resistance in antiretroviral therapy (ART) failure patients, a few studies have distinguished and contrasted between newly diagnosed HIV infection and ART Failures in rural areas [10, 12, 14]. The HIV prevalence in rural China remains severe [16], we assume that the source of newly diagnosed HIV infection come from ART failure patients, molecular network analysis can provide biological non-bias evidence.

## Material and methods

Patient and Public Involvement: Study participants or the public were not involved in the design, or conduct, or reporting, or dissemination plans of our research.

### Study site

With the coverage of sentinel surveillance improving [1] and with a population of 800,000 [17], rural City A ranks one of the top five cities in the number of people living with HIV/AIDS (PLHIV) in Chengdu. District B, City C, and City A are geographically adjacent, facing similar problems of HIV infection continuously increasing.

### Sample

The sample size of newly diagnosed HIV infection was calculated according to the following formula:

$$n=\frac{\mu_{\alpha }^{2}p(1-p)}{\delta^{2}}$$

Where n is the sample size, δ is the allowable error, αis the test level, and P is the sample rate which refers to network access rate of the molecular network in this study. Based on the pilot study results, we take P = 60%, α = 0.05, μ_α_(two-sided test) = 1.96, δ = 0.1×P = 0.1×0.60 = 0.06, calculate n = 256, consider the 20% information incomplete rate, and expand the sample to 320 persons (256/80%). From April 2019 to February 2021, 315 persons were newly diagnosed HIV infection in City A. Due to the failure of amplification of 6 samples of gene, and insufficient amplification length of 1 sample of gene, a total of 308 newly diagnosed HIV infection were included for molecular network analysis.

All ART failure patients from City A, District B and City C during 2017 and 2019 were included, which were 215 cases in total, and 5 cases were excluded (3 cases were due to incomplete basic information of the local AIDS prevention and control information system (ACPCIS), and insufficient amplification length of 2 samples of gene). The main reasons for the failure of antiviral treatment include poor compliance, adverse drug reactions and adverse drug reactions.

**Fig 1:**
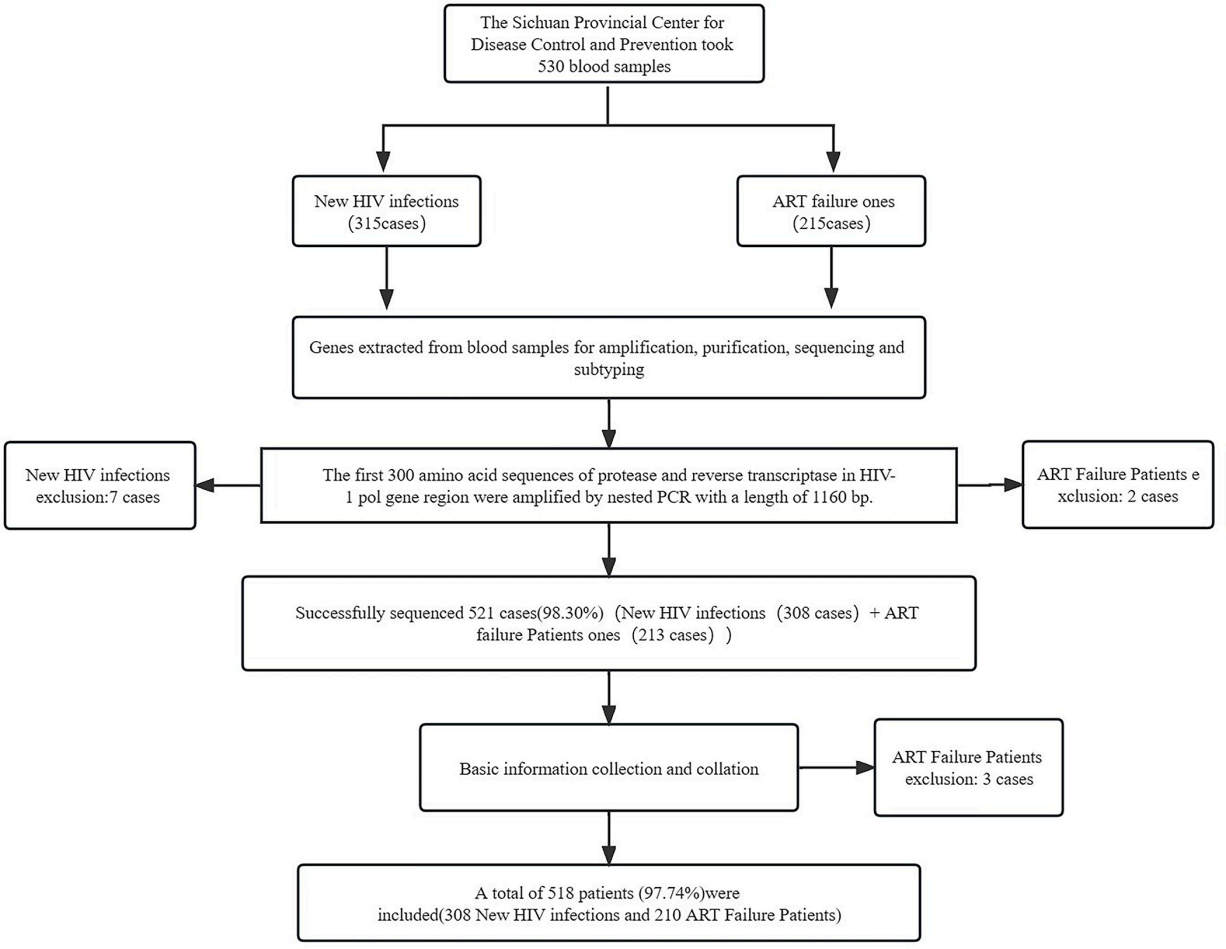
Study inclusion process.

Therefore, a total of 530 cases were included in this study, of which 521 (98.30%) were successfully amplified, and 518 (97.74%) patients were finally included (3 cases were due to incomplete basic information of the local ACPCIS, and insufficient amplification length of 3 genes), including 308 newly diagnosed HIV infection and 210 ART Failure patients. The inclusion flow was showed in Fig 1.

### Data collection

At the time of their first diagnoses, 3-5ml whole blood was collected with 8ml vacuum tube containing EDTA, centrifuged at 1500-3000rpm for 10 minutes within two hours of collection, transferred plasma (0.5ml/tube, 2 tubes) to polypropylene tubes, and collected one tube of lymphatic enrichment liquid. The separated plasma and lymph were enriched and stored at -70°C for delivery. According to the national biological specimen transportation management requirements, the samples were sent to the AIDS Confirmation Center Laboratory of the Sichuan CDC under freezing conditions, and samples’ demographic information, infection routes, viral load and other information were obtained from the local ACPCIS.

The viral nucleic acid was extracted and purified by PCR amplification. According to the kit instructions (Roche Mag NA Pure LC total nucleic acid separation kit), the automatic virus nucleic acid extraction instrument (Mag NA Pure LC system, Roche, Branchburg, NJ) was used for determination [18]. The first 300 amino acid sequences of protease and reverse transcriptase in HIV-1 pol gene region were amplified by nested PCR with a length of 1160 bp [19]. The amplified product with a band at 1200 bp was sent to Beijing Bomaide Gene Technology Co.Ltd. for purification and gene sequencing. The sequencing primers were performed according to the HIV drug resistance monitoring strategy and detection technology [18].

Firstly, we used sequence analysis software Sequencer 4.9 to edit, and cleaned up and spliced the sequences according to the sequence determination and sub-type classification, and then used the software Bioedit to calibrate the sequence and compare and analyze the results with the Los Alamos HIV Database reference sequence [20]. Secondly, we used Fastree 2. 1. 8 and Figtree 1. 4. 2 to construct the evolutionary tree and determine the genotype, and beautify to make a view. Finally using software Run HyPhy 2.2.4 and Cytoscape 3.6.1 [21], TN93 was selected as the molecular network model, MEGA7 was used for phylogenetic evolution, NJ method was used, and GTR was selected as the model. we selected the gene distance value with the largest number of network clusters as the most appropriate threshold to construct a molecular network.

#### Measures

In univariate analysis and multivariate analysis, the following variables are combined into a unified variable due to the small number of cases: nationalities were divided into two categories: “Han” and “other” ethnic groups including “Yi” (1,0.19%), “Qiang” (1,0.19%), “Lisu” (1,0.19%), and “Tibetan” (1,0.19%). As for the occupation, “workers”(10,1.93%), “cadres and staff”(3,0.58%), “seamen and long-distance drivers”(2,0.39%), “public place attendants”(4,0.77%), “commercial services”(11,2.12%), and “catering and food industries”(5,0.97%)were merged into “employment”, “household chores and unemployed” (68,13.13%),“retired personnel”(17,3.28%),student (2,0.39%) and “other occupations” were(10,1.93%) merged into “other”. CD4+ T lymphocytes at the first time of their diagnosis were divided into three intervals of “<200”, “200~349” and “≥350” [22]. Among the strain subtypes, CRF55_01B sub-type (6, 1.16%) and C sub-type (2, 0.39%) were merged into “other” subtypes. “Homosexual transmission” refers to transmission among MSM.

Node refers to an PHLIV or a virus sequence. Degree refers to the number of edges connected between nodes in the network indicating the number of potential communication partners. The higher degree value, the greater risk of communication is. Based on quartiles, degrees were divided into four categories: 1, 2, 3–6, and 7 or more. Two or more nodes are connected to form a cluster, and at least one node is connected to construct a propagation network as a cluster [11, 23]. Studies have shown that concurrent relationship increases the probability of HIV transmission exponentially, and two or more associations indicate that there is a concurrent transmission relationship [24]. One sample connected with two and more nodes was defined as a “high-risk transmitter”, one or less node as “low-risk transmitters”.

Network access rate was calculated as the following:

$$Network\;access\;rate=\frac{Sequence\;in\;the\;cluster}{total\;sequence}*100\%$$

The institutional review boards (IRB) from Sichuan center for disease prevention and control (CDC) approved the protocol. Written informed consent was waived due to no epidemiological investigation were involved. The basic information was obtained from ACPCIS and no specific survey information was involved. This study was conducted in accordance with the Declaration of Helsinki.

#### Statistical analysis

SPSS 19.0 was used for statistical analysis. *x*^2^ test or Fisher’s exact probability method was used for univariate analysis, and the independent variables with *P<0*.*05* in the univariate analysis were included in the binary logistic regression analysis.

## Results

### Basic information

Among the 518 cases, 384 (74.13%)were males; 338(65.25%) age ≥ 50 years old; 514 (99.23%) were Han nationality; 241(,46.53%) were mainly primary school graduates; 286(55.21%) were married; 386(74.52%) were mainly farmers; 360 (69.50%) were mainly in City A; 473 (91.31%) were heterosexual transmission; 320(61.78%) were CRF01_AE sub-type and 240(46.33%) the first CD4+ T cell count were <200 cells/μL, details are showed in Table 1.

**Table 1:** Demographic basic information(n = 518).

Variable	Classification	N	%
Gender	Male	384	74.13
	Female	134	25.87
Age type	18–49	180	34.75
	≥50	338	65.25
Ethnicity	Han	514	99.23
	Minority	4	0.77
Education	Illiteracy	49	9.46
	Primary school	241	46.53
	Junior high school	158	30.5
	High school or technical secondary school	41	7.91
	College degree and above	29	5.6
Marital status	Unmarried	75	14.48
	Married	286	55.21
	Divorced/Widowed	157	30.31
Profession	Farmer	386	74.52
	Worker	35	6.76
	Other	97	18.72
Place of residence	City A	360	69.5
	District B	98	18.92
	City C	60	11.58
Route of infection	Heterosexual transmission	473	91.31
	Homosexual transmission	41	7.92
	Other	4	0.77
Strain sub-type	CRF01_AE	320	61.78
	CRF07_BC	149	28.77
	B	30	5.79
	CRF08_BC	11	2.12
	Others	8	1.54
First CD4+T cell count	<200	240	46.33
	200~	164	31.66
	≥350	103	19.89
	Unknown	11	2.12

### Genotyping statuses

**Fig 2:**
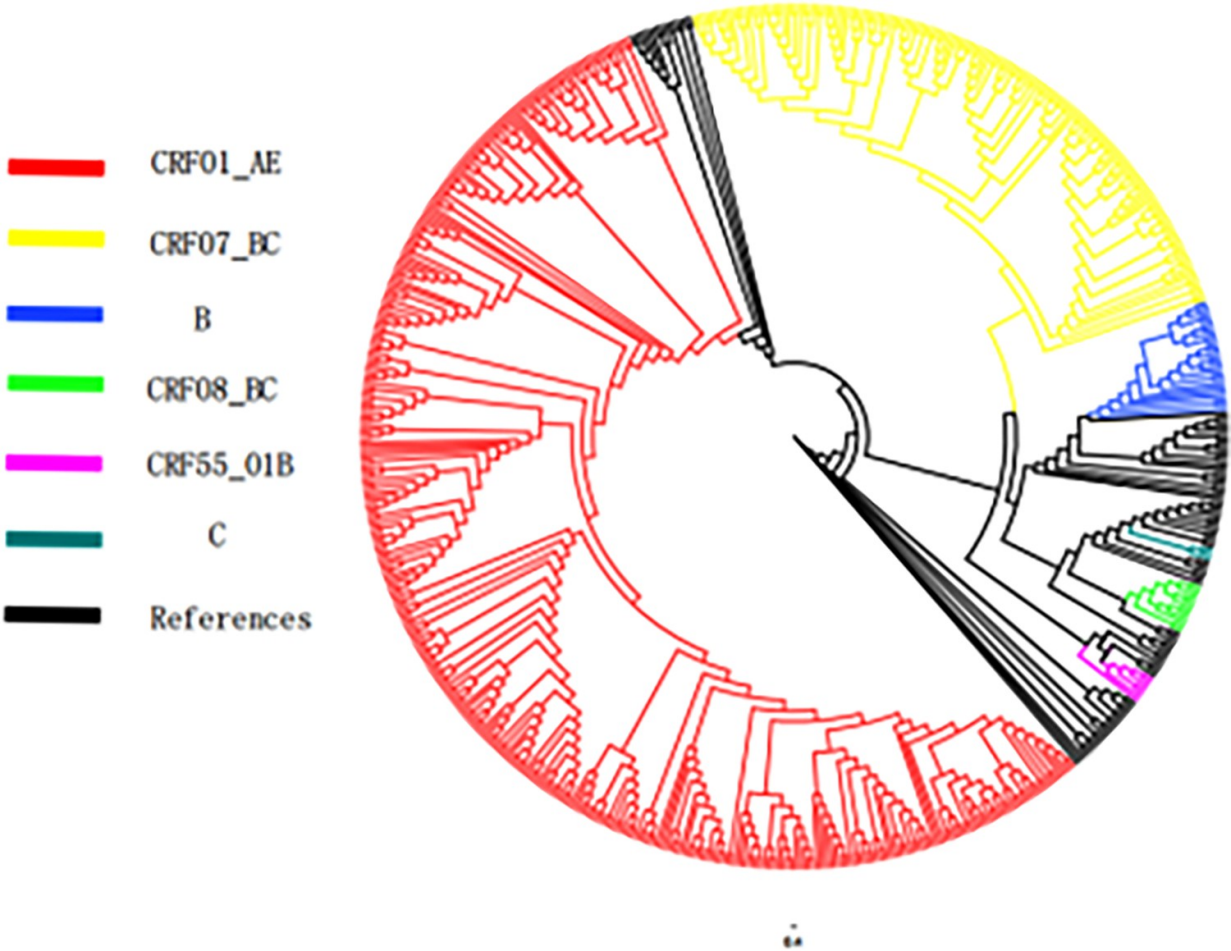
Systematic evolution analysis diagram of HIV-1 pol gene sequence.

Among 518 successfully amplified sequences, 6 strains including CRF01_AE (320,61.78%), CRF07_BC (149,28.76%), B (30,5.79%), CRF08_BC (11, 2.12%), CRF55_01B (6, 1.16%) and C (2, 0.39%), details are showed in Fig 2.

### Transmission characteristics of HIV-1 molecular networks

**Fig 3:**
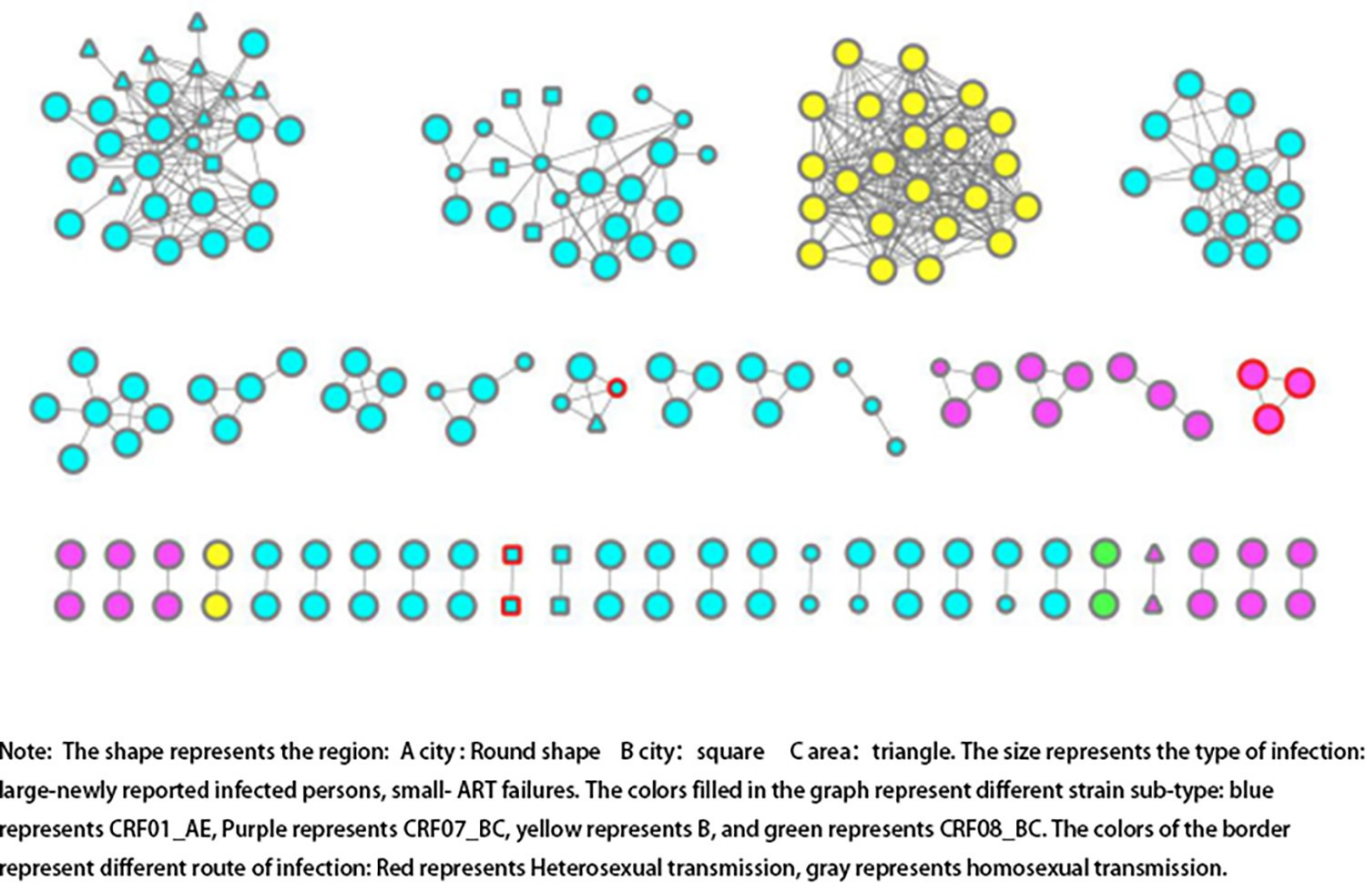
Molecular network transmission map of newly diagnosed HIV infection and ART failure patients in A city, B district and C city (n = 186). Note: The shape represents the region: A city: Round shape B city: square C area: triangle. The size represents the type of infection: large-newly reported infected persons, small-ART failures. The colors filled in the graph represent different strain sub-type: blue represents CRF01_AE, Purple represents CRF07_BC, yellow represents B, and green represents CRF08_BC. The colors of the border represent different route of infection: Red represents Heterosexual transmission, gray represents homosexual transmission.

The molecular network is constructed with the gene distance 0.1%-1.5% [19], in this study, the threshold was 0.8%. A total of 186 sequences entered the final molecular network, forming 42 propagation clusters, and the network access rate was 35.91%. There were cross-infection between newly diagnosed HIV infection and ART failure patients. There were 133 cases of CRF01_AE sub-type forming 28 clusters with a network access rate of 41.56% (133/320), 26 cases of CRF07_BC sub-type forming 11 clusters with a network access rate of 17.45% (26/149), 25 cases of B sub-type forming 2 clusters with a network access rate of 83.33% (25/30), and 2 cases of CRF08_BC sub-type, forming 1 cluster with a network access rate was 18.18% (2/11). Details were showed in Fig 3.

### Distribution of degree among different subtypes in molecular network

54 of 133 (40.6%) in CRF01_AE sub-type group, 16 of 26 (61.54%) in CRF07_BC sub-type and 2 of 2 (100%)in CRF08_BC sub-type group belonged to 1 degree, meanwhile 23 of 25 (92.00%) in B sub-type belonged to 7 and above degree. The difference between CRF01_AE, CRF07_BC, B and CRF08_BC sub-type was statistically significant*(P<0*.*05)*. Details were showed in Table 2.

**Table 2:** Distribution of degree among different sub-types.

Degree	CRF01_AE	CRF07_BC	B	CRF08_BC	*P* (fisher exact test)
1	54(40.6)	16(61.54)	2(8)	2(100)	<0.001
2	19(14.29)	10(38.46)	0(0)	0(0)	
3–6	33(27)	0(0)	0(0)	0(0)	
7 and above	27(20.3)	0(0)	23(92)	0(0)	
total	133(100)	26(100)	25(100)	2(100)	

### Analysis of risk factors for molecular network access

A total of 112 cases (21.62%) were at high risk of transmission. Univariate analyses showed that there were statistical differences in the transmission risk of patients with different ages, education levels, marital status, profession, infection types, residences and strain sub-types *(P<0*.*05*), and there were no statistical differences in the transmission risk of patients with different genders, different transmission routes, nationalities and first CD4+ T (*P>0*.*05*).

Logistic regression analysis showed that and the risk of ≥50 years (OR = 2.474) of age is higher than that of ages 18–49 years, the risk of the CRF07_BC sub-type (OR = 0.174) is lower than that of the CRF01_AE sub-type, and the risk of the B sub-type (OR = 6.698) is higher than that of the CRF01_AE sub-type, the risk of the District B (OR = 0.077) is lower than that of City A, and the differences are statistically significant (P<0.05), details See Table 3.

**Table 3:** Binary logistic regression of high-risk transmission risk factors (n = 518).

Group	Category	B	Sig.	Exp (B)	95% of EXP(B) C.I.	
					Lower limit	Upper limit
Age	≥50 vs .18–49	0.906	0.017	2.474	1.177	5.203
Education	Elementary school vs. illiterate	-0.013	0.974	0.987	0.448	2.172
	Junior high school graduation vs. illiterate	-0.503	0.281	0.605	0.242	1.508
	High school or technical secondary school vs. illiterate	-0.567	0.444	0.567	0.133	2.421
	College degree and above vs. Illiterate	-0.547	0.664	0.579	0.049	6.829
marital status	Married vs. Unmarried	-0.089	0.867	0.915	0.323	2.59
	Divorced/Widowed vs. Unmarried	0.461	0.394	1.585	0.549	4.577
Profession	Working vs. Farmer	-0.358	0.624	0.699	0.167	2.924
	Other vs. Farmer	-0.198	0.603	0.82	0.389	1.73
Type of infection	ART Failure vs. Newly diagnosed HIV infection	-0.72	0.077	0.487	0.219	1.081
Strain sub-type	CRF07_BC vs. CRF01_AE	-1.746	0.000	0.174	0.085	0.36
	B vs. CRF01_AE	1.902	0.000	6.698	2.548	17.606
	CRF08_BC vs. CRF01_AE	-	-	-	-	-
	Others vs. CRF01_AE	-	-	-	-	-
Place of residence	District B vs. City A	-2.563	0.002	0.077	0.016	0.376
	City C vs. City A	-0.115	0.84	0.892	0.293	2.711

Note:—The results are not shown in the regression equation due to small sample.

## Discussion

This study showed that there are 6 sub-types of HIV-1, of which the dominant sub-types are CRF01_AE (320,61.78%) and CRF07_BC (149,28.76%), which are in consistent with the previous molecular epidemiology results both in Sichuan Province and in China [25–28]. Among them, the CRF01_AE strain subtype is the first HIV-1 circulating recombinant strain. During the transmission process, it recombines as a mother strain to form a recombinant strain, which has stronger genetic diversity tolerance. Recent studies have shown that CRF01_AE spread across provinces in clusters grow rapidly and thus spread widely [29]. CRF07_BC is a subtype of B/C recombinant strain. Studies have shown that the co-receptor of CRF07_BC is CCR5 after entering cells. Compared with viruses with X4 tropism, the disease progresses more slowly, which is beneficial to its spread [30].We also found out B (30,5.79%), CRF08_BC (11, 2.12%), CRF55_01B (6, 1.16%) and C (2, 0.39%). These sub-types have been reported in different regions of China before [26, 27].

There are cross-infection between newly diagnosed HIV infection and ART failure patients. 4 of 6 HIV-1 subtypes entered the network, and the transmission routes included homosexual transmission and heterosexual transmission. Sub-network of B sub-type was only composed of newly diagnosed HIV infection in City A. Sub-networks CRF01_AE and CRF07_BC sub-types were all composed of both newly diagnosed HIV infection and ART Failures in A, B and C cities.

The results of multivariate analysis showed that the risk of the CRF07_BC sub-type (OR = 0.174) is lower than that of the CRF01_AE sub-type, and the risk of the B sub-type (OR = 6.698) is higher than that of the CRF01_AE sub-type to enter molecular network. This study showed that the network access rate of sub-type B was as high as 83.33%, of which 92% was associated with 7 and above. Existing studies have shown that the network access rate and the degree in molecular network communication can be used to evaluate and predict the degree of transmission risk, and the higher the degree of relevance is, the easier it is to become a “super transmitter” [31–34]. The first HIV strain found in China is B sub-type [35], some studies have shown that the gene threshold of B sub-type is relatively large between 0.01–0.02 [31], and the molecular epidemiological survey in Sichuan Province has shown that the prevalence of sub-type B strains is a long-term infection [36] and in a low-prevalence infection state [12, 13]. This study shows that the molecular network of sub-type B is highly clustered, only composed of newly diagnosed HIV infection in City A. The median age of at their HIV infection diagnosis is 61 (range: 18–78) years, and the median value of CD4+ T lymphocytes at the first time of their diagnoses is only 235cells/μL (30–435). Therefore, there are two possibilities of infection of B sub-type. The first one is B sub-type strain may be a recent infection and has a strong infectiousness. The second possibility is that that this group has been early infected but not detected. Therefore, it is necessary to strengthen the long-term follow-up and dynamic detection of the traceability investigation of the B sub-type patients in City A to prevent its spread and cross-infection.

The results showed that the risk of ≥50 years of age (OR = 2.474) is higher than that of age 18–49 years. The World Health Organization(WHO) reported that the number of HIV infection among elderly accounted for 25% of the new report in 2020, and the elderly have the highest annual growth rate of HIV infection in China [37]. previous reports in Sichuan Province have also shown that the proportion of HIV infection among the elderly is increasing [38]. Due to the lack of enough HIV-related knowledge and self-protection awareness among the elderly, high-risk sexual behaviors with low condom use result out HIV infection among the elderly people easily, and the transmission is increasing [27, 38, 39].

This study has certain limitations. The transmission relationship is inferred based on genetic distance, it is impossible to determine its actual transmission direction and dynamic change relationship. Therefore, it is necessary to combine social network investigations to trace the source of HIV-infected persons, to identify true transmission relationships and transmission direction, to finally verify that our hypothesis that the transmission route is ART failures→newly diagnosed HIV infection, and provide a better scientific basis for precise prevention.

## Conclusion

In summary, our research results show that the source of HIV infection in Sichuan is diversified and the transmission network is complicated. Prevention and management of HIV infection should focus on strengthening the long-term dynamic testing of the elderly population of sub-type B infection in City A and the elderly in the whole area to prevent its transmission and cross-infection.
